# Physician-reported characteristics, representations, and ethical justifications of shared decision-making practices in the care of paediatric patients with prolonged disorders of consciousness

**DOI:** 10.1186/s12910-023-00896-y

**Published:** 2023-03-07

**Authors:** Vinurshia Sellaiah, Federica Merlo, Roberto Malacrida, Emiliano Albanese, Marta Fadda

**Affiliations:** 1 Faculty of Biomedical Sciences, Università della Svizzera italiana, Via Buffi 13, 6900 Lugano, Switzerland; 2 Institute of Public Health, Faculty of Biomedical Sciences, Università della Svizzera italiana, Via Buffi 13, 6900 Lugano, Switzerland; 3 Sasso Corbaro Foundation, Bellinzona, Switzerland

**Keywords:** Pediatrics, Prolonged disorders of consciousness, Ethics, Shared decision-making, Switzerland

## Abstract

**Background:**

Despite consensus about the importance of implementing shared decision-making (SDM) in clinical practice, this ideal is inconsistently enacted today. Evidence shows that SDM practices differ in the degree of involvement of patients or family members, or in the amount of medical information disclosed to patients in order to “share” meaningfully in treatment decisions. Little is known on which representations and moral justifications physicians hold when realizing SDM. This study explored physicians’ experiences of SDM in the management of paediatric patients with prolonged disorders of consciousness (PDOC). Specifically, we focused on physicians’ SDM approaches, representations, and ethical justifications for engaging in SDM.

**Methods:**

We used a qualitative approach to explore the SDM experiences of 13 ICU physicians, paediatricians, and neurologists based in Switzerland who have been or were involved in the care of paediatric patients living with PDOC. A semi-structured interview format was used and interviews were audio-recorded and transcribed. Data were analysed through thematic analysis.

**Results:**

We found that participants followed three main decision-making approaches: the “brakes” approach, characterized by maximized family’s decisional freedom, though conditional to physician’s judgment regarding the medical appropriateness of a treatment; the “orchestra director” approach, characterized by a multi-step decision-making process led by the main physician aimed at eliciting the voices of the care team members and of the family; and the “sunbeams” approach, characterized by a process oriented to reach consensus with family members through dialogue, where the virtues of the physician are key to guide the process. We also found that participants differed in the moral justifications sustaining each approach, citing the duty to respect parental autonomy, to invest in an ethics of care, and to employ physicians’ virtues to guide the decision-making process.

**Conclusion:**

Our results show that physicians come to perform SDM in different ways, with several representations, and distinct ethical justifications. SDM training among health care providers should clarify the ductility of SDM and the several ethical motivations underpinning it, rather than insisting on the principle of respect for patient’s autonomy as its only moral foundation.

**Supplementary Information:**

The online version contains supplementary material available at 10.1186/s12910-023-00896-y.

## Introduction

Clinical practice standards guiding decision making have moved significantly in recent decades from a paternalistic model to one based on the ethical principle of respect for patient autonomy and, more recently, to shared decision-making (SDM) [1]. SDM is described as an active dialogue between patient and physician with the goal of reaching a mutual understanding and agreement on a treatment plan. The process of SDM involves the comprehensible delivery of evidence-based medical knowledge and the exchange of preferences, values, and expectations between patient and physician [2]. The practice of SDM is recommended by many guidelines and is usually integrated in the academic training of physicians. Evidence shows that SDM is linked to better treatment adherence, disease coping, clinical outcomes, and quality of life [3].

Yet, evidence showed that SDM is interpreted by physicians in various ways and inconsistently realized in the clinical context, mostly in terms of the degree of patient’s involvement and amount of information shared with patients [4]. Studies showed that, beyond the level of patient involvement (who makes the decision), also the quality of SDM (how the decision is made) is highly variable [5]. This variance can be explained by different factors such as physicians’ own conceptualization of SDM and its perceived relevance, limited time for dialogue, or the ambiguity about which decisions do require the enactment of SDM [6]. In addition, SDM is often taught as a step-by-step and monolithic approach grounded in the communication exchange between patient and physician, which does not fully reflect what happens in the every-day clinical practice, where communication is more fluid and unpredictable [7, 8]. Following this rationale, the concept of “purposeful SDM” has been proposed, arguing that the way SDM is practiced should vary according to the clinical problem and that the general SDM approach needs situational adaptation [9]. An empirical understanding of how SDM is practiced in the clinical setting is vital to reducing the gap between ideal and actual practices and advancing medical education on decision-making. The context of paediatric patients living with prolonged disorders of consciousness (PDOC) presents an ideal setting for studying SDM. SDM in paediatrics involves an additional layer of complexity since there are three parties involved in decisions: the child, parent(s) and the clinician. It needs to be noted that the state of the science on SDM is mainly based on literature on the rational for SDM in competent adult patients. The ethical rationale for involving parents in shared proxy decision-making is not identical to the rationale for involving competent patients in SDM. In the former case, it must be argued why parents should have the authority to determine which strategy is best to consider the patient’s best interests (or their presumed will), whereas in the latter case, it must be argued why physicians should respect the patient’s autonomy. Some have argued that when it comes to decisions regarding children with conditions limiting their ability to participate in decision-making, decisions are made by their parents and clinicians *for* the child rather than *with* the child [10, 11]. Although SDM is espoused as an ideal to facilitate family-centred care, research is immature on how this approach is realized in the setting of care for children living with severe disorders of consciousness. This setting is characterized by the lack of documented patient’s preferences on possible medical decisions and difficulties in constructing the patient’s biography, the complexity in understanding how to sustainably manage the patient in the long-term, the many actors involved, and the need for careful decision-making during high-risk acute events [12]. A previous qualitative study by Vemuri et al. with paediatricians caring for children with life-limiting conditions (LLC) showed that paediatricians framed their decision-making approach as SDM, but when they described their roles and responsibilities these were aligned with an intentional physician-led approach [13]. The authors also found that paediatricians alluded to their instinct to protect the child from harm and the parents from psychological burden and possible ongoing harm of making a very difficult decision as main ethical justification for such an approach. The aim of this study was to explore how physicians used SDM when caring for paediatric patients living with PDOC as well as how they represented and morally justified their preferred approach. In particular, we aimed at identifying which aspects of SDM are more meaningful than others for our sample (what our participants place an emphasis on when reflecting on their preferred SDM approach) rather than focusing on a given aspect (or set of aspects) of the SDM approach on which to elicit our participants’ opinion.

## Methods

Between 2019 and 2020, we conducted a qualitative study using semi-structured, individual interviews to explore physicians’ decision-making approaches when managing paediatric patients with PDOC. This article describes the results of an exploratory secondary analysis of a sub-sample of the interviews collected. The results of the primary analysis of the full corpus of the interviews are published elsewhere [14]. Both the primary study and the present analysis were guided by an interpretive (hermeneutic) phenomenological approach [15]. The method and reporting followed the Consolidated Criteria for Reporting Qualitative Research [16].

### Recruitment and sample

In the primary study, a sample of 19 Italian-speaking physicians employed in or retired from either an intensive care unit (ICU) or paediatric, internal medicine, or neurology department in Switzerland was recruited through a snowball sampling technique. Potential participants were asked to confirm they had experience in managing paediatric patients with PDOC. Participants were contacted by three of the authors (FM, RM and MF) by e-mail or phone and invited to participate in the interview. During the call and in the email, we explained the nature and scope of the study, and reassured potential participants that their reports would be kept confidential and only portions would be shared in anonymized form for publication purposes. In the present analysis, we included 13 out of the 19 participants (68.5%) interviewed in the primary study. The interviews selected were conducted in the second phase of the primary study that served as data source for the present secondary analysis. This second phase specifically targeted the topic of SDM. The first six interviews conducted in the primary study (i.e., those conducted in the first phase) did not address SDM in details and did not provide sufficient value to the analysis to justify their inclusion. The reason for selecting Italian-speaking physicians based in Switzerland as participants for this study is twofold. The first one is linguistic. One of the interviewers in the primary study, also co-author in the present investigation, did not master other languages except Italian at the moment of data collection. Even if the interviews could be conducted by the second interviewer in another language, this would have introduced problems in the analysis as transcriptions would have needed to be translated, losing some meanings. The second reason is the convenience sampling approach used in the primary study. We selected participants based on our knowledge of their past/current clinical activity.

### Data collection

The interviews included in the present study were conducted between July 2019 and February 2020, and lasted approximately one hour. Data were collected in Switzerland, either on the phone or in person at the participants’ office. The interviewers (FM and MF) were trained in qualitative research and had substantial experience in conducting individual interviews. Both interviewers employed open-ended questions and a non-judgmental approach to the interview. A semi-structured interview guide (Appendix 1) was developed based on the literature and expert consultation. The interview addressed the main clinical decisions involving patients with PDOC, and participants’ preferred approaches for decision-making. After participants’ oral informed consent, interviews were tape-recorded and transcribed verbatim. Transcripts were not returned to participants for comment and/or correction.

### Data analysis

Two coders (VS and MF) employed an inductive thematic analysis to extract meaningful themes from the data, with a particular focus on participants’ preferred decision-making approach(es). The analysis proceeded as follows. One by one, each interview transcript was independently analysed to extract meaningful quotes. Subsequently, the two coders convened and discussed their independent evaluation. As the analysis of the interviews proceeded, constant reference was made to the previously analysed interviews. Starting from the third interview, a conceptual map was created to identify and describe emerging patterns (e.g., similar decision-making approaches that could be categorized under the same label). Following the analysis of all transcripts, a new round of analysis allowed the coders to refine the conceptual map and to improve the categorization of the emerging approaches to SDM. The coders identified convergences and divergences, clarified meaningful distinctions between approaches, and isolated relevant, reported metaphors for each approach. Finally, the coders verified that each approach was substantiated by the data, associated to the respective moral justification(s) reported by participants, and supported by relevant quotes. Data saturation was discussed among the two coders and found to be reached after ten interviews. We did not ask participants to provide feedback on the findings.

## Results

### Participants’ characteristics

Of the 13 included physicians, 3 identified themselves as women. The sample included two intensive care physicians, seven paediatricians, and four neurologists. Eleven participants were employed, while two were retired at the moment of the interview. The mean age was 55.5 years (range = 45–71), and the average years of experience was 28.8 years (range = 19–40). Participants’ characteristics can be found in Table 1.

**Table 1:** Characteristics of study participants (N = 13)

Characteristic	Value
Gender, n (%)	
Female	3 (23%)
Age, years	Mean = 55.5 (SD = 8.6, range = 45–71)
Specialty, n (%)	
Intensive care	2 (15%)
Pediatrics	7 (54%)
Neurology	4 (31%)
Occupation, n (%)	
Employed	11 (85%)
Retired	2 (15%)
Experience, years*	Mean = 28.8 (SD = 7.3, range = 19–40)

*Years of experience are counted since obtaining medical degree

### Participants’ preferred decision-making approaches

**Fig. 1:**
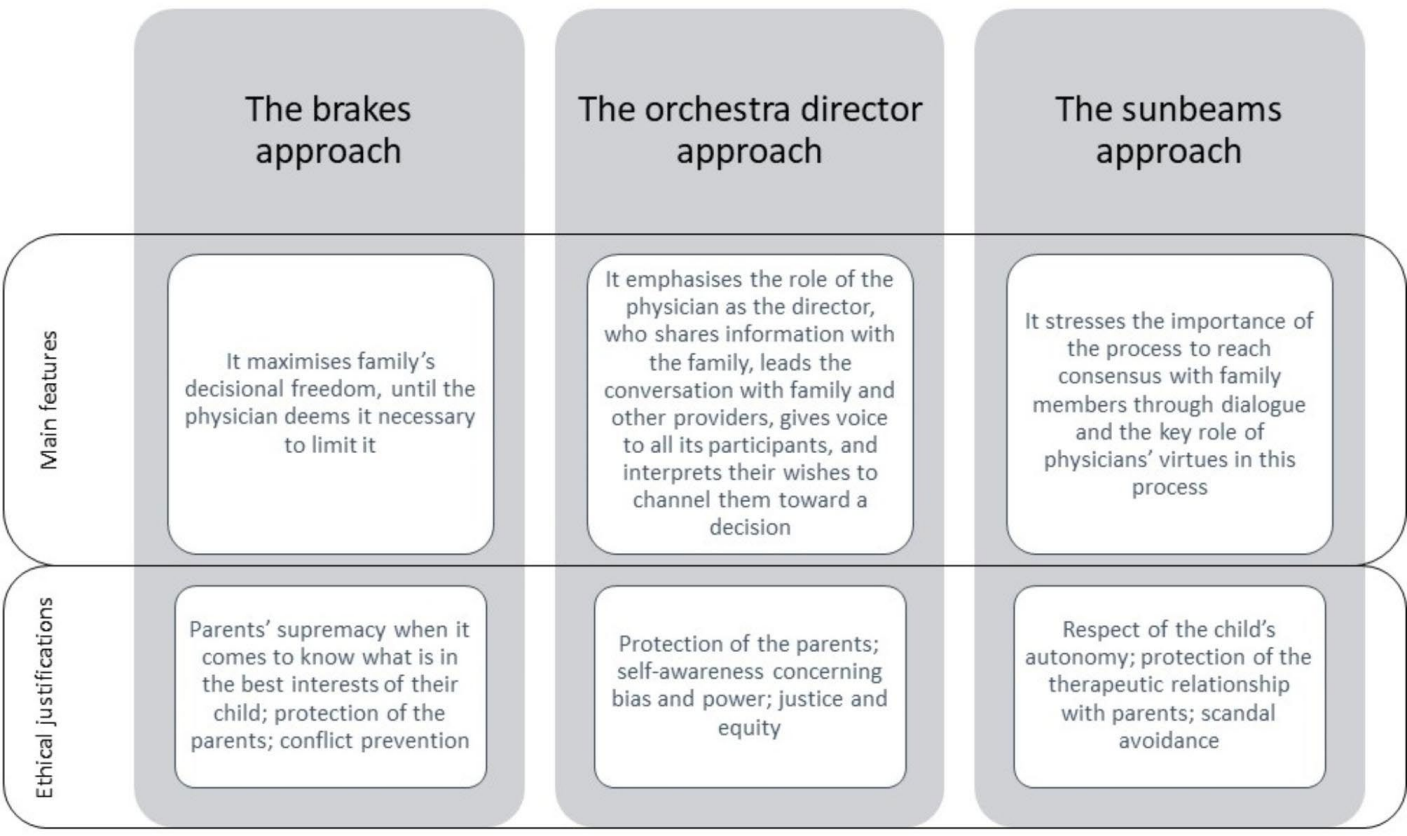
Thematic map of the research findings

When we asked participants to describe their preferred decision-making approach when managing paediatric patients living with PDOC, all stated they favoured the SDM approach. However, when we prompted a more detailed description of their approach, and asked them to report which aspects they attributed more importance to, they reported a number of preferences, attitudes, and behaviours which could be grouped in three main distinct approaches emphasizing one or more elements of the SDM approach. The first approach is the “brakes” approach, where the family is given maximum freedom until the physician deems it necessary to limit it, justified with the principle of respect for parental autonomy. The second is the “orchestra director” approach, where the physician provides information, elicits preferences, interprets desires, proposes a course of action to the family and moderates the discussion between them and the care team to reach a decision. This approach was justified with the duty to care and to maximize the care investment. These two concepts were described as distinct by our participants. The “duty to care” was described as referring to the moral and professional obligation to provide therapeutic care to the patient, which is in line with the principle of beneficence. The duty to maximize the “care investment” was described as referring to caring for interpersonal relationships and care or benevolence as a virtue, which is more in line with a feminist ethics of care normative framework. The third approach is the “sunbeams” approach, where the physician employs his or her experiences and qualities (the sunbeams) to invest in a consensus-oriented communication with the family, grounded in virtue ethics. Notably, results showed that participants emphasized clearly distinct aspects of SDM as key elements of their approaches: participants supporting the brakes approach emphasised the distribution of power between the two parties involved; participants supporting the orchestra director approach stressed the importance of the tasks of the physician in the decision-making process participants supporting the sunbeams approach placed an emphasis on the description of the goal of the SDM process (reaching consensus). Furthermore, two participants preferred approach overlapped with more than one of the approaches described below. Most participants reported being aware of the shift in clinical decision-making from a paternalistic to a SDM model, but only two were aware of the diversity in the SDM approaches. Common to all approaches was the perceived importance of recognizing that each patient and family contexts are different and need to be addressed in their uniqueness and specificities. All participants agreed that physicians should make an effort to know the child as a person as much as possible, the family context and the number of children, including the weight attributed to each member, and create a relationship before deciding on how to proceed. A thematic map of the research findings is available in Fig. 1.

### The brakes approach

Six out of thirteen participants reported a preference for an approach that maximises family’s decisional freedom, until the physician deems it necessary to limit it. Participants referred to this approach using the metaphor of car driving: the family is in the driver’s seat, while the physician sits next to them and holds the brakes to stop the car when it goes beyond a certain point:

*[…] It’s always the family that has the steering while in their hands and it’s up to the clinician to try to understand and to be able to enter a very close relationship with the family. (Interview 1, ICU physician)*

*We do what the family says and answer questions if the family needs information, but the decision, at least from my perspective, is fully up to the family. (Interview 9, neurologist)*

*Sometimes there are parents who have thought things through, but that decision should not be made by the parents. I don’t think it is possible for the doctor to do something if he or she is not convinced, even if the parents want him or her to do it. If the parents wanted Exit for the child, the doctor would have to be very convinced, and it also depends on what he or she experiences, to be able to say that it is going to be done. […] I would say that doctors, when they talk to each other, set their limits and try to tell these limits to parents or the institution. (Interview 8, paediatrician)*

These participants saw their role as someone who should accompany and be present next to the family during the patient journey:

*There are some families, for example, who are not ready right away. Especially in traumatic brain injuries, because up to the day before you were at home quietly and the next day, I can’t tell them that there is nothing more we can do, so you have to give time for people to mature. So, it’s almost a process of accompanying the family members as well. (Interview 1, ICU physician)*

*Yes, I have to accompany. Of course then questions always come up, like: but doctor what would you do if it was your child, if it was up, if it was down. But I never give a precise answer, it depends. (Interview 11, paediatrician)*

For these participants, the patient’s family needs to be cared for as much as, if not more than, the patient:

*Sometimes I was told that it was too early for a certain thing, although aware of the situation, although aware that the chances of getting out of it are zero, today I cannot decide to stop the therapy, because for the family members it is still too early. (Interview 1, ICU physician)*

Participants identified instances where the family’s psychological, emotional, and moral interests would take priority over a non-suffering child. For example, one participant explained that providing life-sustaining treatment to a child at the end of life should be seen by the care team as a final present for the family, an act of sincere caring and compassion even if the care team does not consider the treatment as being in the best interest of the child:

*Perhaps this is the last gift we can give to the family, that there is an accompaniment according to their timelines. […] We have to think about ensuring […] a good death, so that the memory the family will keep throughout their lives is not a bad memory. So, we try as much as possible to make that moment as peaceful as possible for the whole family. (Interview 1, ICU physician)*

*I can think of one case where the family was not ready to let the patient go and so we went a little further, we put the PEG, so as not to create conflict, but this*

*prolonged his life in hospital, with infections, so with discomfort also in the people who were caring for him. It is important to understand that when you are trying to content a family member who is not ready for a funeral, this results in a sick person who is passively kept alive, cleaned, and moved. (Interview 10, neurologist)*

According to participants, pulling the brakes and refusing the requested treatment is only justified when it would cause substantial suffering or when the demands of the family are clearly at odds with the interests of the patient:

*It is up to the care team to defend [the child] and make sure he or she will no longer suffer. (Interview 12, ICU physician)*

*The parent is often not the one who makes the “negative” decision. Now we know that probably in certain situations, doing a therapy of comfort is, ethically speaking, the best solution for that individual to not make him/her suffer any further. Here, from an ethical point of view, this is a dilemma because how do you decide if the parents cannot decide? Does the physician decide? No, the physician does not decide, it’s not like the physician is the god on earth who decides. I think it is important to go hand in hand on the same path with the family and showing them the right direction. (Interview 5, paediatrician)*

Participants justified their approach legally and ethically. From a *legal* point of view, they reported that the therapeutic representative should have the final say in the patient’s decision:

*If I, from a legal point of view, told the mother that the next pneumonia will not be treated anymore but she instead absolutely wants to treat it, from a legal point of view I am obliged to treat it, because she is the health care surrogate decision maker and the one who interprets the patient’s wishes. (Interview 1, ICU physician)*

They also reported that physicians may be legally constrained by national or hospital policies to refuse to provide treatments that are disproportionate compared to the goal of care or excessively burdensome for the patient:

*[It depends on the] general framework within which one knows one can move, i.e. if I work at the [name of a hospital] in Rome I can never perform passive euthanasia, but if I work in Holland I do, I can even help paediatric patients to die. So where do I work at the moment? Am I in a facility that limits me? Where are these limits? […] Once I have that clear, everything else is enough. I don’t necessarily need more. (Interview 11, paediatrician)*

From an *ethical* perspective, they justified this approach with several claims. First, they recognized parents’ supremacy when it comes to know what is in the best interests of their child:

*[…] Consider starting from the fundamental principle that a child’s father and mother always want the best for their child. If you start from this principle, you do the best thing for the child in agreement with the parents, because you start from the principle that the parents want the best. (Interview 5, paediatrician)*

Second, they argued that the family should not be the ultimate decision-maker, especially when it comes to limiting treatment, because they may live for years with the psychological consequences and regrets of their end-of-life decisions:

*Parents, in my experience, never want to feel guilty for a “negative” decision for their child. It is very rare to find, because it’s probably the nature of the parent in this situation […]. It is probably the nature of the parent to always do everything possible for their child. (Interview 5, paediatrician)*

*It is clear that the mother could not be the one to make this decision, also to take away this burden and guilt, so the feeling of guilt in case something happened. (Interview 8, paediatrician)*

Third, they argued that this approach may help physicians to anticipate parents’ decisions regarding the point to which they would like to go in terms of treatment and procedures. According to one participant, being aware that the physician will likely limit parental decisions when he or she deems them clinically or ethically inappropriate, anticipating such decisions may facilitate preventing conflicts between families and care teams:

*What we do is we sit down with several people where one of the people is represented by the intensive care physician or anaesthesiologist, and we talk to the family members to try to understand how far they want to go. […] Sometimes we find it hard to reach it [consensus]. Who wins in these cases? The one who wants to move forwards. […] So, winning in this sense, but it also happened to me that this word was appropriate because the level of conflict was such that the final outcome is almost a victory for one of the members. It depends on the degree of conflict, but whenever it occurs it is easy for one to follow the path of the one who is more conservative and not the one who says “stop”. (Interview 9, neurologist)*

### The orchestra director approach

Four participants reported to follow a multi-step decision-making process. The first step is to explain to the family both the physiological processes and the consequences of long-term home care for the parents and the patient’s siblings:

*It is very important to understand and to describe what keeping a child alive means and what the consequences of long-term home care are: it means intensive care at home with alarms going off every two or three minutes, even during the night; it means that there is no guarantee of having a nurse 24h a day. You also have to consider the impact on other children, on siblings who are often forgotten. (Interview 2, paediatrician)*

Participants argued that this should be done in a language that the family can understand, and acknowledging the emotional turmoil they may be experiencing:

*[…] the family members need to be informed about the situation because there can be misconceptions. And you have to understand that they are in a very emotional state and might not always understand as well as we think, and you have to have the patience to go back and talk with them and to adapt the language to a level that allows for adequate communication and that is not always easy. (Interview 3, neurologist)*

In the next step, the physician (usually the one caring for the patient) makes an effort to understand the family’s position, eliciting hopes, concerns, and what matters to them:

*I don’t have a good rule in saying that it needs to be done this way or that way. I try to discuss and understand the various expectations that are on the table, try to let the thoughts shine through and to see what other scenarios might look like, then you try to balance everything out. (Interview 4, paediatrician)*

The third step is represented by a discussion oriented to find an agreed course of action, with the physician leading the conversation, giving voice to all its participants, and interpreting their wishes to channel them toward a decision. Participants represented this approach with the metaphor of an orchestra, where the physician acts as the director:

*So this is the final part of the decision making process, which is a decision made together while someone has to take the role of the orchestra director. (Interview 3, neurologist)*

Different stakeholders (specialists, nurses, family members) play their instrument, and the physician tries to harmonize the interests of all participants. Key to this approach is physicians’ emotional investment in the care of the patient:

*Affectivity is one of the most important aspects and what we can give in these cases is mainly presence, comfort, and all of these attributes. (Interview 4, paediatrician*)

Participants reported that physicians should embrace an empathic approach, recognize the difficulties families experience in caring for their child, in being physically separated from him/her, and in seeing their expectations being unmet:

*When I first started, sometimes I wondered about the point of [parents, who were] insisting [to continue treatment], even in the presence of infections, whether to treat them or not. [Now] I put myself on the other side and think about those who are going through this situation and are not ready to separate. (Interview 4, paediatrician)*

Participants following this approach believed they ought to lift a moral burden from parents, prevent them from experiencing guilt, and take the moral responsibility of the decision after listening to their preferences:

*The decision should be made by the medical team, making a proposal to the parents, who must almost object if they disagree, but not in a paternalistic way. It is important to spare the parents the burden and responsibility of deciding to let the child die, but clearly you never make anyone die because it’s illegal. (Interview 2, paediatrician)*

Participants recognized the importance of becoming aware of one’s biases at multiple levels, projections, the sources of one’s intuitions, and come up with strategies on how to mitigate them. First, physicians should become aware of their own biases and how these could possibly influence the trajectory of clinical decisions in order to mitigate them:

*Maybe it’s a little bit philosophical, but we often believe that the child shows us [what it needs], but sometimes it can be our projection onto the child and you have to be very self-critical and differentiate. (Interview 2, paediatrician)*

*Often what I see is, that doctors are very much influenced by their own opinion about it. Their opinion has quite an influence, so if they see a catastrophic situation, if they are of the opinion that this condition will lead to an unacceptable clinical condition, this message is conveyed to the family members, who in a tragic situation, are definitely influenced by it. (Interview 6, neurologist)*

Second, they should become aware of their power in the delicate relationship with patients and family members and learn how to avoid abusing it. Third, they should advance their awareness of justice and equity issues, and invest in health advocacy, particularly for those patients that receive less attention because they are less “attractive” to policy and funding:

*These children do not have a lobby, [their disease] is not sexy, they are not hairless oncology children with big eyes that are always on advertisements for research in oncology, those are children who talk. Deciding when life is or is not worth living is an extremely difficult and extremely dangerous decision for the society as a whole. (Interview 2, paediatrician)*

Finally, physicians should be aware of the pressure that society and religion place on families of children living with PDOC (e.g., the idea of sanctity of life) and the power of media in driving public opinion:

*It is very difficult to make the right choices in terms of the right amount, in the right way from a therapeutic point of view as well as with regard to the community. So when there is a demand for doing as much therapy as possible, sometimes you have situations that are very loaded, where you provide so much therapy, which also puts so much strain on the targeted person in this case. On the other hand, the society reads this as something good. (Interview 4, paediatrician)*

*When she died, she was no longer a little girl but a woman, but when they were showing all those pictures in the media… […] she had been in that state of illness for about 15 years, so she had become a cachectic person who was dying and that had nothing to do with the pictures of the smiling little girl. The message they wanted to get across however was, that a young, smiling girl was being killed. As if she was being shot in the head while she was skiing. (Interview 6, neurologist)*

Participants justified this approach with the “duty to care”, referring to the moral and professional obligation to provide therapeutic care to the patient, but also with their duty to maximize their “care investment”, referring to caring for interpersonal relationships and care or benevolence as a virtue. To them, investment meant “being invested in care”, not necessarily with a specific therapeutic goal, and not necessarily to achieve clinical progress:

*I remember patients with children, who will never make progress […]. So, I say, what matters is not the progress but doing as much as possible for the greatest gift that is the child. The parents have a child with big problems and severe disabilities and so as a parent, you want to invest as much as possible, not taking into account that there is a whole world spinning around you. (Interview 4, paediatrician*)

### The sunbeams approach

Four participants reported an approach strongly focused on the process to reach consensus with family members through dialogue. For these participants, consensus can and should be reached at all costs, even in the most difficult situations:

*The first thing, the keyword I have always lived for and searched for is “consensus’”. Always. (Interview 7, paediatrician).*

According to these participants, consensus is always possible provided that the physician engages in a process aiming to reach the “core” of the relationship with the patient’s family, i.e., what makes us universally human. To achieve this, physicians need to have a certain human maturity, they need to have strong wisdom qualities, which a participant referred to as “sunbeams”. These are necessary virtues for a physician to “shine” and bring clarity on the situation:

*There are people who radiate; [I can say that] rays come out of their sun, and these raysmake them [these people] interesting. […] A human maturity comes out. (Interview 7, paediatrician).*

The most important factor is the maturity of the physician(s) engaged in the process aiming at consensus. Physicians need to have a certain human maturity to use this approach well. This maturity is linked to a personal journey, in the sense that life experiences are key to shape the physician in a mature human being:

*Because I am made of those experiences; I was made by those experiences. (Interview 7, paediatrician)*

For the same participant, trustworthiness is also a key element in the process to reach a consensus with the family. To this end, possessing human maturity also means being and becoming someone who can be trusted by the family:

*The word trust means... you are mature and I consider you mature, therefore I think I can trust you. The word maturity is not very intellectual, it comes from the gut, it is very intuitive. (Interview 7, paediatrician)*

Key to this approach are involving the extended context of the child in the decision-making process (not only the family), understanding the meaning that the family attaches to the child’s illness, and considering the invasiveness and the consequences of the intervention (not only on the future, but also on the past):

*For example, let’s take the insertion of a PEG, so the possibility of feeding the patient who is not known whether he or she is able to feed himself. This is a decision that has a big bearing on the future of this person and that reinterprets the past in very different ways, and therefore it is a big decision. Much more than the decision of when and how to make this action, which from a medical-surgical point of view is a simple medical action. (Interview 13, paediatrician)*

Two participants placed a strong emphasis on the life story of the child as an important element to drive the process aimed at consensus. In this sense, the physician should make an effort to acquire a biographical account of the child from different perspectives. This should include information on the activities the child valued or used to carry out, but also the relationships that were meaningful to them:

*One has to think about what the person did when he or she was well, so also with the person with a disability, what the person did when he or she was well, so what he or she did willingly, and then whether the current situation can be approached in the same way as the pre-existing situation, which does not have to be the current condition, which for someone can be bad, but for the person can be beautiful. (Interview 10, neurologist)*

*We need to find out about the historical and biographical elements that brought this child to this particular situation, as well as the affective relational context surrounding this child and the institutional context. (Interview 13, paediatrician)*

Participants placed a stronger importance on the decision-making process than the decisional outcome. They considered this to be a tiered approach where little steps are done at each time, re-evaluated regularly, and the most important aspects are addressed first:

*Two very different outcomes can be equally the result of good deliberation; the important thing is that it is good deliberation and that there is a basis for good deliberation. Not a utilitarianism that says “this is what it takes for six-year-olds”, I don’t see it that way, but rather a good deontological approach that says that there are partial aspects that we consistently evaluate and that give a kind of view of the situation and that when compared with each other and weighed carefully can lead to a process that must be the basis for any decision. (Interview 13, paediatrician)*

*Let’s start tackling the main ones, see how it goes and then get back to it. It doesn’t all have to come out perfect straight away, it can be a piece, that is those points that I think are the main risk. What is the problem I am most at risk of dealing with? Then if a new problem arrives, you deal with it in a standard way, and when you realise there’s something new, you deal with it a little bit ... It depends: one can put together a list of thirty questions, and you can do it like that: the same form you do in a quarter of an hour and, maybe, another one does it in three years. (Interview 11, paediatrician)*

They justified this approach with the argument that it brings clarity to a murky context where the patient’s importance is likely to decrease with reduced autonomy or lack thereof:

*It is really important that there is clarity in all aspects of the context […] What is very important is that there is clarity on everything that is at stake; once there is this clarity, then it can be said: this is the opinion of the parents, who may have a type of opinion filtered, for example, by the desire for filiation, by the desire and disappointed expectations of having a healthy child, by what are their projections on the future, the projections on other figures with whom they are confronted, etc. This is a context that should be read and respected in this way, so it should not be somehow thrown into the fray of opinions of a care team that has a mission, as it is serving the clinical needs given by a situation of a child’s illness and somehow the kind of expectations and projections are different, are coloured differently. They are not to be put in opposition, just as the presumed will, the right to life, the dignity of the person, etc. are not to be put in opposition, which when mixed together make a big mess. (Interview 13, paediatrician)*

In addition, they reported that this process fosters the relationship of therapeutic alliance between the paediatrician and the family. In a consensus-based approach, this relationship is crystallized and it becomes the place where the child keeps living even after his or her death.

*The choice should always be individualised and an alliance with the family and parents should be sought and a compromise reached. (Interview 10, neurologist)*

*After his death, his mother often came to see me and had this perception of presence; the perception of something that was not finished in the same way as the paper I threw away just now, before you came in. This experience plays a decisive role. (Interview 7, paediatrician)*

Finally, one participant also reported that this approach helps to find a solution to avoid the scandal:

*The ideal is to find a solution to avoid ending up in the newspapers, or those unpleasant things, where it doesn’t go away and then it has to be a judge to decide and maybe that’s not exactly ideal. (Interview 11, paediatrician)*

## Discussion

We carried out a secondary analysis of semi-structured, individual interviews conducted with ICU physicians, paediatricians, and neurologists to explore their SDM practices, representations, and ethical justifications when it comes to manage paediatric patients with prolonged disorders of consciousness. We found that participants understood the concept of SDM in several ways both conceptually and morally.

A first finding is that participants reported to invest in different aspects of SDM. At its core, SDM is a “process in which clinicians and patients work together to select tests, treatments, management, or support packages, based on clinical evidence and the patient’s informed preferences” [17]. Our participants, however, offered a different answer to the question of what makes decision-making “shared”. For participants following the brakes approach, practicing SDM was a matter of maintaining the delicate power relationship between families and physicians, and swinging between bestowing unconditional decisional freedom to families and re-establishing their authority when families’ decisions were no longer acceptable. For participants following the orchestra director approach, SDM was a matter of fairness of the decision-making process. These participants emphasized the importance of the extent that the process is fair by including everyone in the conversation, voicing everyone’s wishes/concerns, increasing physician’s awareness and reducing biases. For those supporting the sunbeams approach, SDM is a matter of engaging in a process aimed at reaching consensus, building relationships (not in terms of power, but to the extent that powerful relationships are able to transcend the child’s life and keep the child’s story alive), and cultivating the virtues of the “good” physician. Other studies highlighted the idea of not only considering the best interest of the child as the centre of the decision but also involving the interests of the family in order to balance the values adequately [18]. In this context, being aware of stressors that families experience, giving them the time to adjust to the situation or protecting the family from guilt or regret over a decision is described in the literature [19]. Limiting options, as proposed in the “brakes approach”, is a strategy that was voiced in another study, where physicians felt obliged to establish limits, particularly when the lack of beneficence of a treatment was evident [20]. The relevance of accompanying parents and family members throughout the whole process and giving them the chance to come to own conclusions is acknowledged in the literature as well. Other studies also emphasized the aim of reaching consensus as one of the key elements of SDM and that creating an alliance between the involved parties is crucial for a successful SDM approach [21]. As pointed out by some of the participants in this study, physicians practicing SDM need to recognize power imbalances and become aware of the biases affecting their own preferences [22]. Few of our participants reported to engage in a distinct set of considerations that is often neglected in paediatrics decision-making, i.e., considerations related to the patient’s biography. Rather than asking what is best for the child/adolescent or what the patient would have chosen, a standard based on the patient’s biographical narrative asks what decision is most consistent with is the patient’s life account [23]. Our findings are novel, as some participants declared to make an effort to extract biographical elements from families’ accounts in order to understand the patient’s identity and make decisions accordingly. Furthermore, there is new insight to each preferred approach and to the values of physicians that uniquely shape their strategies when caring for children with PDOC. Specific elements and their relevance in performing SDM could be extracted and explained from analysing the interviews, which allowed us to see beyond the general and broad description of SDM. Results showed that not only values of patients and families, but also the values and moral justifications of physicians reshape the process and outcome of SDM in a significant way. Our results are also in line with the arguments that SMD involves work that is cognitive, emotional, and relational [22]: SDM cannot be reduced to a simple transaction where information is shared and preferences are accounted for – it requires a significant investment in the relationship with patients and families, in one’s professional identity, and in one’s emotional commitment to care.

The second finding is that participants reported different moral justifications for each approach identified. The state of the art on SDM is mainly based on a vast literature on the ethical rational for SDM in competent adult patients. The ethical rationale for involving parents in shared proxy decision-making is not identical to the rationale for involving competent patients in SDM. While, in the former case, it must be argued why parents should have the authority to determine which strategy is best to consider the patient’s best interests (or their presumed will), in the latter case it must be argued why physicians should respect the patient’s autonomy. Along this line, there is widespread agreement that patients have both a right to know about their medical situation and to have their preferences considered the basis for professional actions involving them [24]. Glynn and colleagues argued that the underpinning principle for SDM risks being overshadowed by arguments that SDM is a method to reduce health care costs or to ration care to patients [25]. Our results showed that participants were aware that the ethical rationales for proxy decision-making in paediatrics differ from those guiding SDM with competent adult. Vemuri et al. conducted a qualitative with paediatricians caring for children with LLC to explore SDM practices and ethical justifications for enacting SDM [13]. In line with our results, they showed that physicians framed their approach as SDM, but when they described their roles and responsibilities these were aligned with an intentional physician-led approach [13]. The authors also found that paediatricians alluded to their instinct to protect the child from harm and the parents from the psychological burden and possible ongoing harm of making a very difficult decision as main ethical justification for such an approach [13]. Our study shows that physicians come to practice SDM with additional moral foundations compared to those identified by Vemuri et al. Some favoured SDM because it ensures respect of *the family’s* autonomy (rather than the child’s), it prevents harm to the child (by ensuring that the best interests of the child are protected), it avoids conflicts with family, and it is in line with what a *good* physician would do. Others justified it with the claim of protecting the family from experiencing guilt or regret and with the duty to maximise the care investment (in terms of relationships and benevolence as virtues). Some defended it because it helps maintain a therapeutic alliance with the family and clarify the context around the child in order to find a balance between conflicting demands for physicians. This result should not surprise, as evidence shows that physicians may prioritize one or more moral concerns over others, such as the fairness of the decision-making process rather than the clinical and ethical outcomes of the decision [14].

### Implications and recommendations

This study has a number of implications for both theory and practice. From a theoretical perspective, our results confirm previous evidence that SDM is conceptually understood in various ways [8, 13]. Since scholars warned of a risk that SDM’s identity as a respectful, empathic and patient-focused approach may become less visible [25], it is important to highlight the many nuances of SDM’s identity perceived by physicians. This study contributes to the debate on narrow and broader conceptions of SDM, their relative strengths and weaknesses, and the necessary trade-offs between different conceptions in order to realize the ethical goals of SDM [26]. Our results showed that it is not only a matter of restricting or maximizing patient or parental autonomy when realizing SDM, but also about attributing roles and responsibilities during the SDM process in ways that do not necessarily alter the level of support of patient autonomy, or orienting the process in a way that either maximises parental freedom, enhances the process’ fairness, or is guided by the relational qualities of the physician.

In practice, our results contribute to the evaluation of SDM practices aiming at understanding their potential long-term consequences for clinical practice. For example, different practices can diverge over questions of whether, when and how it is appropriate to recommend a particular treatment or challenge a patient’s expressed preferences. Our results also point to some limitations in the teaching of SDM in medical education. As previously suggested, SDM training should allow physicians to recognize the need to adapt the SDM approach to each case, and importantly on how to justify it ethically and clinically [9]. Furthermore, SDM education should overcome the predominance of a step-wise approach [27, 28]. Such an approach forces SDM into a highly structured approach that leaves little space to adapt in a way that is best in line with what he or she values. Our study showed that there is not one best way to enact SDM, but that this could be flexibly practiced according to different modalities and grounded in various ethical justifications.

### Limitations

This study had some limitations. First, we cannot exclude that some participants may have reported their opinion in a socially desirable manner. To reduce this bias, a non-judgmental approach was used by the interviewer during the collection of the data. Second, since participants were self-selected, they could have already been interested in the topic of the investigation and have reflected in depth on it prior to the interview, which may not be the case of physicians who did not take part in the study. Finally, since our study represented an enquiry into a specific setting and involved a small sample, there is no automatic generalisation in the statistical sense beyond the cases involved, even if our participants were instrumentally selected and their reports studied to stand for the category of cases to which they belong. However, offering a thick description of our participants’ reports supports a naturalistic generalisation of our research findings embedded within readers’ personal and unique experiences [29, 30]. We believe that the reader is sufficiently informed about the cases described in our study to make judgements about the extent to which our findings may be applicable to their own situations. We acknowledge that certain legal and cultural conventions may shape decision-making for paediatric patients with prolonged DOC among Italian-speaking physicians based in Switzerland. While no data are available on the prevalence of end-of-life decisions through passive euthanasia in the paediatric PDOC setting in Switzerland to estimate whether these are made more or less often in this country compared to other regions in Europe or to other countries, what makes our study interesting is the specific sample that we interviewed. We included in our study participants who were based in Switzerland, a country where passive euthanasia is legal, but who – being Italian (native) speakers – are likely to be culturally exposed to or influenced by Italian culture. In Italy, no form of euthanasia is currently allowed, and euthanasia is similar to voluntary murder even if the patient is consenting and, therefore, considered a crime [31]. Familiarity with the Italian cultural, social, and legal context may have shaped decision-making attitudes, behaviours, and ethical justifications among our sample.

## Conclusion

Although shared decision making is held up as an ideal decision-making standard with a specific ethical goal, it is used to describe decision-making practices that are different in their nature, representation(s), and moral justification(s). Our participants were aware of the difficulties of enacting SDM in the context of the care of paediatric patients living with PDOC, and they responded in different ways, bringing their own conceptions and moral foundations. In order to genuinely realize SDM and its ethical aim, SDM training for physicians, nurses, and social workers should highlight the nuanced nature of such a decision-making approach, the several ways in which it can be achieved, and the various ethical justifications underpinning it.

## Electronic supplementary material

Below is the link to the electronic supplementary material.

Supplementary Material 1: Interview grid

## Data Availability

The datasets generated during and analysed during the current study are not publicly available due to the fact that data contain potentially sensitive information that may allow identification of the study participants, but are available from the corresponding author on reasonable request.
