# Supplementary material for: Physician-reported characteristics, representations, and ethical justifications of shared decision-making practices in the care of paediatric patients with prolonged disorders of consciousness
Source: BMC Med Ethics. 2023 Mar 7;24:19. doi: 10.1186/s12910-023-00896-y (PMC9993525; doi:10.1186/s12910-023-00896-y)
Supplement: Supplementary file 1 — Supplementary Material 1: Interview grid [file 12910_2023_896_MOESM1_ESM.docx]

**Appendix 1.** Interview grid

| Theme | Question |
| --- | --- |
| Definitions |  |
| Persistent Vegetative State (PVS) | - How would you define the Persistent Vegetative State? |
| Minimally Conscious State (MCS) | - How would you define the Minimally Conscious State? |
| Comparison between the two conditions | - What are the main differences between the two conditions? |
|  |  |
| Main clinical decisions in adult patients |  |
| PVS | - Which are the main clinical decisions when managing adult patients in PVS? |
| MCS | - Which are the main clinical decisions when managing adult patients in MCS? |
|  |  |
| Main clinical decisions in children patients |  |
|  | - Which are the main clinical decisions when managing pediatric patients with PDOC? |
|  |  |
| Criteria for decision-making and approaches |  |
|  | - Why do you think different clinical decisions are made in similar clinical situations? |
|  | - Are there guidelines for clinical decision-making with PVS and MSC adult/pediatric patients in your hospital (or in other hospitals in which you worked)? If so, what role do they play in your decision-making process? |
|  | - How would you describe your decision-making process approach? Can you think of factors that play a role during your decision-making process? Which of these factors do challenge such guidelines? |
|  |  |
| Shared decision-making |  |
|  | - Think about the role of patients’ family members on your clinical decision-making process: what comes to your mind? - How do you share your decision with family members? |
|  |  |
| Quality of life/health status |  |
|  | - What do you take into consideration when evaluating the child’s/adolescent’s quality of life prior to the trauma/event? |
|  | - What role does the initial health status of the child/adolescent play in your evaluation of his/her quality of life prior to the trauma/event? |
|  | - How would you define the quality of life of a child/adolescent in PVS/MCS from birth? |
|  |  |
| Etiology |  |
|  | - What role does etiology play on your decision-making, compared to prognosis? |
|  | - Think about different etiologies: what difference does it make if the patient had traumatic or non-traumatic (hypoxia) brain injuries from birth? |
|  |  |
| Diagnosis |  |
|  | - What role does diagnosis play on your decision-making, compared to prognosis? |
|  |  |
| Prognosis |  |
|  | - What do you take into account when making a prognosis? |
|  |  |
| Treatment |  |
|  | - What do you consider to be an “aggressive treatment”? |
|  | - How do different treatments affect PVS/MCS patients’ quality of life? |
|  | - When deciding for treatment, would it make a difference whether the child/adolescent has been in PVS/MCS since birth? If so, how? |
|  |  |
| Distributive justice |  |
|  | - Do you do anything to ensure that management/treatment costs are equally distributed? If so, what? |
|  |  |
| Autonomy |  |
|  | - What do you think is the best way to respect the autonomy of a child/adolescent in PVS? What do you do? - What do you think is the best way to respect the autonomy of a child/adolescent in MCS? What do you do? - Who should be the guardian of this autonomy? Who, in fact, is? |
